# How social support influences learned helplessness in lung cancer patients: the chain mediation role of individual resilience and self-efficacy

**DOI:** 10.3389/fpsyg.2024.1436495

**Published:** 2024-09-05

**Authors:** Jingui Huang, Yumei Shi, Yuemei Chen, Ling Tang, Zhaoli Zhang

**Affiliations:** ^1^Department of Medical Oncology, Chongqing University Cancer Hospital, Chongqing, China; ^2^Department of Nursing, Chongqing University Cancer Hospital, Chongqing, China

**Keywords:** lung cancer, learned helplessness, social support, individual resilience, self-efficacy

## Abstract

**Background:**

Social support, which is a crucial external resource for cancer patients, was demonstrated to be a positive predictor of learned helplessness (LH). But it is far from clear whether and how social support decreases the LH in cancer patients. The purpose of present study is to detect the association between social support and LH and the role of individual resilience and self-efficacy in mediating this relationship.

**Methods:**

The convenience sampling method was utilized. From August 2022 to February 2024, a total of 537 lung cancer patients (*M*_age_ = 60.25 years, SD_age_ = 9.85 years) from five tertiary hospitals in one municipalities (Chongqing), and two provinces (Sichuan and Yunnan) were recruited, among which 389 were males and 148 were females. LH, social support, individual resilience, and self-efficacy were assessed by using standard scales. A structural equation model was constructed employing AMOS 23.0 to examine the interrelationships among social support, individual resilience, self-efficacy, and LH of lung cancer patients.

**Results:**

A total of 537 lung cancer patients were finally included. Social support, individual resilience, and self-efficacy were positively related to LH (*r* = −0.299 to −0.451, *p* < 0.01). The mediation model revealed that the direct effect of social support on LH was significant (*β* = −0.407, *p* < 0.001). Besides, social support could also affect LH through three pathways: (1) the mediating effect of individual resilience (*β* = −0.075, *p* < 0.001); (2) the mediating effect of self-efficacy (*β* = −0.060, *p* < 0.05); (3) the chain mediating effect of individual resilience and self-efficacy (*β* = −0.011, *p* < 0.05).

**Conclusion:**

The results indicate that social support alleviates lung cancer patients’ LH, and that individual resilience and self-efficacy mediate the correlation between social support and LH. Besides providing adequate social support, intervention strategies built on individual resilience and self-efficacy should be applied to reduce LH in lung cancer patients.

## Introduction

According to the latest statistics, lung cancer ranked first in the incidence and mortality in Chinese men and women, with approximately 1.06 million new cases and 730,000 deaths in 2022 (Zheng et al., 2024). Patients diagnosed with lung cancer have to bear the physical symptoms induced by the disease and continuous therapies (Duan et al., 2022), but they must also grapple with the psychological burden derived from social alienation (Takemura et al., 2022), financial toxicity (Liu M. et al., 2023) and so on. In the course of long-term treatment, patients frequently experience a sense of powerlessness with respect to managing their disease status, which may lead to the development of learned helplessness (LH). Huang et al. (2024) also stated that lung cancer patients are subject to LH due to the physical symptoms, intensive treatments, and poor prognoses that accompany the condition. LH was defined as a psychological condition typified by a sense of powerlessness or self-abandonment, which manifests itself in cognitive impairments, diminished motivation, and emotional dysregulation when individuals perceive a discrepancy between their behavior and outcome (Camacho et al., 2013). LH was proved to be negatively associated with self-management in maintenance hemodialysis patients (Xie et al., 2023a). Patients with high LH lack confidence in their ability to manage their disease and lives effectively, and are susceptible to negative anticipations, and consequently, they are more likely to abandon their efforts or persevere for a shorter duration (Xie et al., 2023b). Worse, LH as a persistent negative psychological state can naturally induce more serious psychological problems, such as cognitive biases, embitterment, anxiety and depression, and even suicidal intention (Xie et al., 2022).

In order to identify more potent strategies for mitigating individuals’ LH, a multitude of studies have been conducted to probe its influencing variables. A recent research has discovered that high social support can provide spiritual and material help for individuals and thus buffer the impact on middle or high school students’ LH (Liu et al., 2024). Social support is described as the emotional, instrumental, tangible, and informational resources derived by individuals from their social network (Schaefer et al., 1981). While it has been verified that social support is an effective variable in mitigating LH, yet there is a dearth of research focusing on lung cancer patients. Social support was the key factor associated with LH, but the mechanisms underlying the association between social support and LH remains obscure. Therefore, the main focus of this research is to determine the extent and manner in which social support mitigates LH in patients diagnosed with lung cancer.

As described above, it is indicated that greater social support was associated with lower LH. In accordance with the psychological stress theory, individuals undergoing stressful events can benefit from social support, which functions as a buffering mechanism to safeguard their physical and mental well-being (Ozbay et al., 2007). A systematic review revealed that the level of fear of cancer recurrence decreased as the social support increased (Lu et al., 2023). Besides that, it is found that social support was negatively related to the degree of depression (Tao et al., 2022), anxiety (Li Q. et al., 2023), psychological distress (Okamura et al., 2023) and illness uncertainty (Wu et al., 2024) among cancer patients. Based upon the previous studies, we propose hypothesis H1: social support negatively predicts LH in lung cancer patients.

In addition to the effects of social support on LH, individual resilience is also closely related to LH (Kang and Zhao, 2020). Resilience is a complex and multidimensional concept that has achieved popularity in positive psychology as a significant human strength due to its impact on psycho-somatic and social health (Sharif-Nia et al., 2024). With the advances in theory, methods, and knowledge, resilience science in psychology matured over the ensuing decades (Masten et al., 2021). Currently, resilience is defined for scalability and integrative purposes as the capacity of a dynamic system to adapt successfully through multisystem processes to challenges that threaten system function, survival, or development, which can be applied to diverse systems, including individuals, families, businesses, communities, economies, or ecosystems (Masten, 2019). In the empirical study, Hughes et al. (2024) found that increases in individual resilience during cancer treatment were associated with reduced symptoms of depression and anxiety. A high positive correlation was also indicated between posttraumatic growth and individual resilience in people with breast cancer (Wan et al., 2023). Resilience serves as a crucial component of positive mental resources, which enhances cognitive processing and positive coping strategies during life adversities (Shi et al., 2021). Hence, individual resilience may negatively predict LH in lung cancer patients.

Furthermore, prior research has indicated that there was a positive correlation between individual resilience and social support (Wang et al., 2024), and social support can positively predict their individual resilience in lung cancer patients (Yin et al., 2022). Resilience is considered a dynamic mechanism that changes over time and can be affected by life circumstances, one’s environment, and situational as well as contextual factors (Masten, 2019). In addition to biological factors, such as gene–environment interactions, personal factors including coping mechanisms and hope, along with environmental factors, particularly social support, collectively determine an individual’s resilience in the face of the cancer experience (Seiler and Jenewein, 2019). So, we propose hypothesis H2: individual resilience mediates the relationship between social support and LH.

Apart from social support and resilience, research has confirmed that more self-efficacy was associated with less LH in oncology patients. Huang et al. (2024) also discovered that a greater level of self-efficacy was correlated with a lower grade of LH in lung cancer patients. Self-efficacy refers to an individual’s level of confidence in achieving a specific behavioral objective, which ascertains the feasibility of implementing the behavior, the required amount of effort during the process, and the duration it can be sustained in the face of setbacks (Bandura, 1977). In accordance with self-efficacy theory (Bandura, 1977), self-efficacy has the ability to regulate individuals’ behavior by influencing their decision-making, perseverance, attitude toward challenges, and cognitive processes. Additionally, Li X. et al. (2023) pointed out that colorectal cancer patients with higher self-efficacy affirmed their ability to perform physical activity, which helped them adhere to regular physical activity and showed a stronger determination to overcome difficulties. Therefore, self-efficacy may negatively and directly predict LH among lung cancer patients. Interestingly enough, a previous study has identified that there is a certain correlation between social support and self-efficacy in cancer patients, and social support can positively and directly predict self-efficacy (Zhang et al., 2023). In accordance with the transactional theory put forth in 1984 (Lazarus and Folkman, 1984), the manner in which individuals respond to stressors is contingent upon their subjective appraisal of the environment and the coping strategies employed in dealing with such events. And the appraisals and coping strategies can be influenced by external circumstances, such as multidimensional social support (Liu S. et al., 2023). On the basis of the transactional theory and previous literature, we put forward hypothesis H3: self-efficacy serves a mediating role between social support and LH.

Meanwhile, a close association between individual resilience and self-efficacy has been verified in prior research. Chinese scholars (Wang R. H. et al., 2023) found that the association of individual resilience with self-management behaviors was fully mediated through self-efficacy in patients newly diagnosed with type 2 diabetes. Furthermore, a cross-sectional study with cadets conducted in Lithuanian indicated that individual resilience exerts a positive effect on self-efficacy (Navickienė and Vasiliauskas, 2023). In terms of the revised model of resilience (De Caroli and Sagone, 2014), individuals with higher resilience tend to seek the “positive side” in challenging situations, and they can harness positive emotions to manage stress and regulate behavior, thus fostering greater self-confidence in facing adversities. Taken together, for lung cancer patients, social support may pose a positive effect on individual resilience, and, thus, promotes self-efficacy and further reduce LH. Naturally, we raise hypothesis H4: individual resilience and self-efficacy have a chain-mediating effect on the relationship between social support and LH in patients with lung cancer.

**Figure 1:**
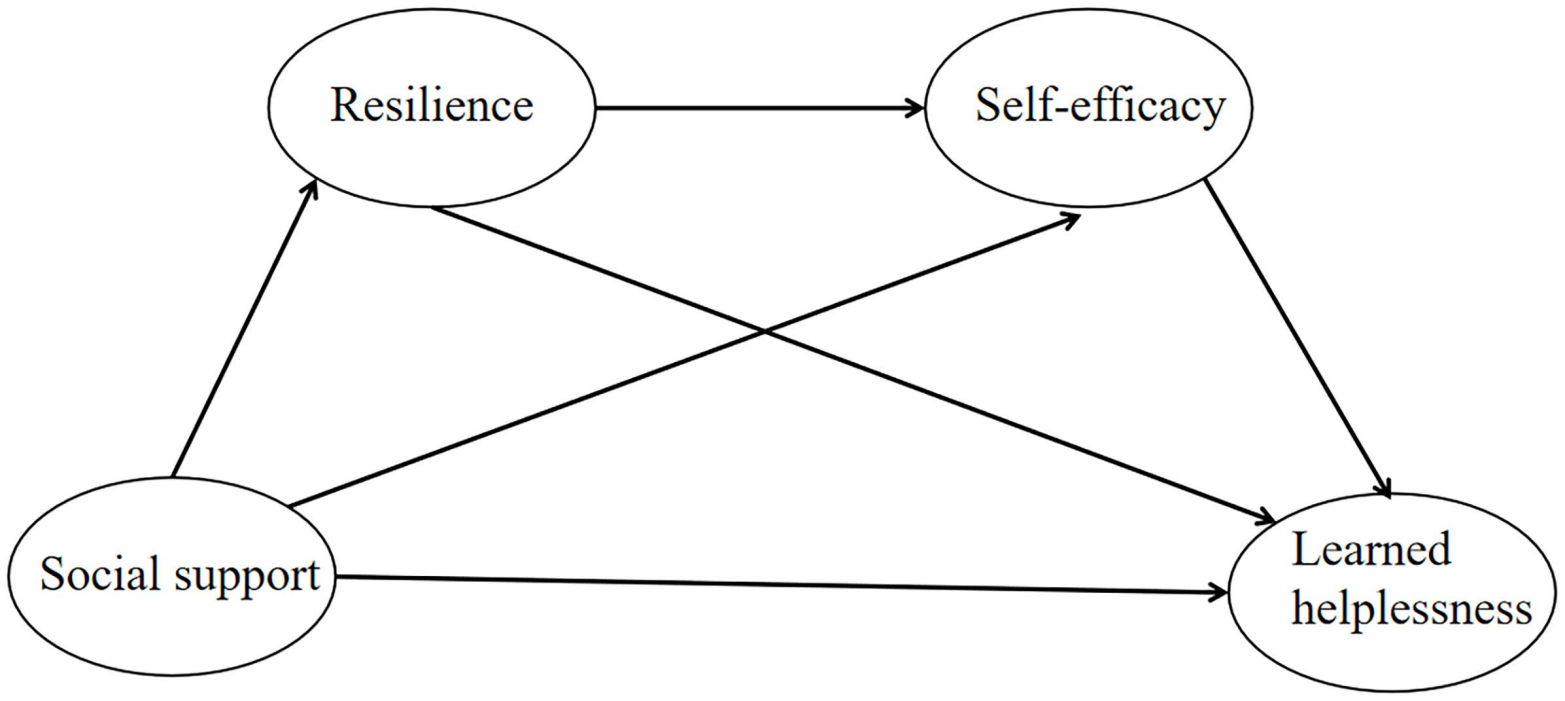
Hypothesized model.

Based on what we currently know, no studies have focused on how social support, individual resilience, self-efficacy and LH interact with each other in Chinese samples of lung cancer populations. By delving into the underlying mechanisms of these factors, we can offer new insights into analysis of the relationship between the four variables, and also empirical supports and scientific evidence for corresponding interventions toward at minimizing the adverse effects of LH on cancer patients. This exploration possesses significant theoretical and practical implications. So, this study aims to examine the associations between social support, individual resilience, self-efficacy, and LH of lung cancer populations and potential mediating effect of individual resilience and self-efficacy via the structural equation modeling (SEM) analysis. By combining the above theoretical model and empirical research, we formulated the following four research hypotheses (Figure 1):

H1: Social support is negatively correlated with LH.

H2: Individual resilience mediates the link between social support and LH.

H3: Self-efficacy mediates the link between social support and LH.

H4: Individual resilience and self-efficacy sequentially mediate the link between social support and LH.

## Materials and methods

### Participants

According to the accessible range of team ability, a total of five tertiary hospitals in southwest China were selected from August 2022 to February 2024, based on convenience sampling method from one municipalities (Chongqing), and two provinces (Sichuan and Yunnan). Questionnaires were distributed in the relevant departments that admitted lung cancer patients (including thoracic surgery department, medical oncology department and respiratory medicine department). The eligibility criteria of participants were: (1) pathologically diagnosed with lung cancer, (2) age ≥ 18 years old, (3) able to speak and write in Chinese, and (4) volunteer to participate. Exclusion criteria: (1) previous history of psychological or psychiatric disorders; (2) suffering from other serious complications. Regarding the sample size required for SEM analysis, it is recommended that a sample size of 100 is poor, 200 is fair, and 300 is good (Tomarken and Waller, 2005). Considering a non-response rate of 10%, the sample size of this study should be greater than 330.

A total of 562 lung cancer patients completed the questionnaires according to the inclusion criteria, and 25 invalid questionnaires were excluded due to consistent patterns of response (12 cases), illogical answers (eight cases), and outliers (five cases). Five hundred and thirty-seven valid questionnaires were obtained, with an effective recovery rate of 95.6%. The mean age of the participants 60.25 ± 9.85 years (range: 26–87), among whom 389 (72.4%) were male and 89.6% were married. Most patients had adenocarcinoma (43.0%), and 245 participants (45.6%) presented with stage IV disease. Other demographic characteristics are summarized in Table 1.

**Table 1:** Participant characteristics.

Variables	Frequency (*N*)	Percentage (%)
**Gender**		
Male	389	72.4
Female	148	27.6
**Education**		
Primary school or below	153	28.5
Junior middle school	231	43
Senior middle school or same level	98	18.2
College or above	55	10.3
**Marital status**		
Married	481	89.6
Others (single/ widowed/divorced)	56	10.4
**Annual per capita household income (¥Yuan)**		
<5,000	321	59.8
5,000–10,000	141	26.3
> 10,000	75	13.9
**Cancer duration (month)**		
<6	212	39.5
6–12	127	23.6
>12	198	36.9
**Pathology type**		
Adenocarcinoma	231	43
Squamous cell carcinoma	162	30.1
Small cell	92	17.1
Big cell	52	9.8
**Tumor stage**		
I and II	142	26.5
III	150	27.9
IV	245	45.6
**Tumor metastasis**		
Yes	261	48.6
No	276	51.4
	**Range**	**Mean (SD)**
**Age**	26–87	60.25 (9.85)

¥, ¥ 1 equivalent to 0.15 United States dollars; *N*, patient number; SD, standard deviation.

### Instruments

#### Learned helplessness scale

Learned Helplessness Scale (LHS), developed by Chinese scholar Wu et al. (2009), was applied to measure LH in lung cancer patients. It has been used in the sample of Chinese lung cancer patients (Huang et al., 2024), breast cancer patients (Li et al., 2024) and college students (Xue et al., 2023). The scale consists of 18 items, containing 2 dimensions (helplessness and despair). Participants rate their agreement with each statement using a 5-point Likert scale from 1 (not at all) to 5 (ideally), with higher total scores reflecting a greater level of LH. The Cronbach’s *α* for the total scale was 0.930, with a split-half reliability of 0.901, and a retest reliability of 0.898 (Wu et al., 2009). For the current sample, the Cronbach’s *α* for the whole scale, helplessness subscale, and despair subscale were 0.865, 0.820, and 0.877, respectively, suggesting excellent reliability. And the KMO value showing validity was 0.912.

#### Perceived social support scale

The Perceived Social Support Scale (PSSS) compiled by Zimet et al. (1990) and adapted to Chinese by Huang et al. (1996) was adopted to test social support of participants. PSSS consists of 12 items, containing 3 dimensions (friend support, family support, and other important support). The items were rated using a 7-point Likert scale, with a response format ranging from “1 = strongly disagree” to “5 = strongly agree,” with higher scale scores corresponding to more social support. Cronbach’s alpha for the Chinese population was 0.88 (Huang et al., 1996). For the current sample, the Cronbach’s *α* for the whole scale, friend support subscale, family support subscale, and other important support subscale were 0.833, 0.842, 0.850, and 0.824, respectively, suggesting satisfactory consistency. And the KMO value showing validity was 0.960.

#### 10-item Connor-Davidson resilience scale

The 10-item Connor-Davidson Resilience Scale (CD-RISC-10) complied by Campbell-Sills and Stein (2007) and revised by Wang et al. (2010) was selected to measure individual resilience in the present sample. Each question was rated on a 5-point Likert scale varying between 0 (never) and 4 (nearly always). The minimum and maximum total scores of the scale are 0 and 40, respectively, with higher scores representing higher resilience. The Chinese version was demonstrated to have good internal consistency (Cronbach’s *α* = 0.91) and test–retest reliability (*r* = 0.90 for a two-week interval) (Wang et al., 2010). In this study, the Cronbach’s *α* for the whole scale was 0.917, indicating excellent internal consistency. And the KMO value representing validity was 0.935.

#### Strategies used by people to promote health

The Strategies Used by People to Promote Health (SUPPH) developed by Lev and Owen (1996) culturally modified by Qian and Yuan (2011) was applied to determine the status of participants’ self-efficacy in this study. SUPPH consists of 28 items, including 3 dimensions (stress relief, positive attitude, and self-decision). Each item was rated on a 5-point Likert scale ranging from 1 (no confidence) to 5 (very confident). The minimum and maximum total scores of SUPPH are 28 and 140, respectively. SUPPH total scores were categorized as low level (28–55), medium level (56–112), and high level (113–140). Cronbach’s alpha for the Chinese population was between 0.849 and 0.970 depending on the sub-dimensions (Qian and Yuan, 2011). For the current sample, the Cronbach’s alpha for the total scale, stress relief subscale, positive attitude subscale, and self-decision subscale were 0.974, 0.953, 0.957, and 0.876, respectively, suggesting excellent reliability. And the KMO value reflecting validity was 0.950.

### Procedure

This study was performed in line with the ethical standards of the Declaration of Helsinki. Approval was granted by the ethics review institution of Chongqing University Cancer Hospital (No. CZLS2022022-A). This is a multicenter cross-sectional survey. Specific procedure is as follows: The research instruments are made into electronic questionnaires for data collection. The research personnel first contacted the head nurses of each department in the investigated hospitals, and explained the purpose, significance and attention points of the study to them. After obtaining their consent and support, the head nurses organized patients to fill in the electronic questionnaire based on the inclusion and exclusion criteria. Before filling in the anonymous questionnaire, the respondents are required to read the informed consent, and they can jump to the official answer page after reading and agreeing to take part in the survey. All questions in the electronic questionnaire are mandatory. If any information is omitted during the response process, the questionnaire cannot be submitted. And the system will automatically display a prompt to complete the required information, ensuring the integrity of the survey. To prevent duplicate data collection, a single IP address and device can only be submitted once. Prior to conducting statistical analysis, invalid questionnaires with identical answers or consistent responses are excluded.

### Data analysis

Epidata 3.1 (Lauritsen and Bruus, 2003) was applied to the entry of data. In order to guarantee that the confirmatory factor analysis of each variable satisfies the statistical prerequisites, it is necessary to conduct the common method bias (CMB) test, multicollinearity test and outliers test prior to data analysis. We used Harman’s single factor test to explore the CMB. Cook’s distances and Variance inflation factor (VIF) were adopted to determine the outliers and multicollinearity, respectively. Continuous variables are described as the ranges, means and standard deviations (SD). In addition, Categorical variables are described as counts and percentages. Pearson’s correlation analyses were selected to assess the correlations of LHS scores with PSSS, CD-RISC-10, and SUPPH scores. SPSS version 23.0 (IBM Corporation, 2015) was utilized for all of the above data analyses. In statistical decisions, all tests of significance were two-tailed with a level of 0.05.

In addition, SEM was computed with SPSS 23 AMOS (IBM Corporation, 2015), which was used to detect the mediating effect and generate an analysis diagram revealing the relationship between LH, social support, resilience and self-efficacy. The significance of the mediating effect was tested via the bootstrap method, with sampling for 5,000 times, and 95% confidence intervals (CI) were computed. If 95% CI did not contain zero, the indirect effect was significant. The model’s goodness of fit was assessed using the following indexes and recommended limits (Chiao et al., 2018): root-mean-square error of approximation (RMSEA) < 0.08; standardized root-mean-square residual (SRMR) < 0.08; χ^2^/*df* < 3; incremental fit index (IFI) > 0.90; comparative fit index (CFI) > 0.95; goodness of fit index (GFI) > 0.90; tucker Lewis index (TLI) > 0.90; normed fit index (NFI) > 0.90.

## Results

### Descriptive statistics

The results showed that the average Learned Helplessness Scale score was 50.31 ± 8.64, with a minimum of 33 points and a maximum of 82 points. And the average score for helplessness and despair is 35.34 ± 6.64 and 14.98 ± 3.04, respectively. The minimum, maximum, mean and SD of other measures are shown in Table 2.

**Table 2:** Descriptive statistics of each measure.

Measures	Dimension	Minimum	Maximum	Mean	SD
LHS	**Total**	33	82	50.31	8.64
	Helplessness	21	60	35.34	6.64
	Despair	6	25	14.98	3.04
PSSS	**Total**	27	84	59.42	10.08
	Friend support	7	28	19.29	3.86
	Family support	8	28	20.53	3.77
	Other support	5	28	19.60	3.81
SUPPH	**Total**	37	140	89.77	21.40
	Stress relief	11	50	31.44	8.22
	Positive attitude	18	75	47.39	11.45
	Self-decision	3	15	9.73	2.63
CD-RISC-10	–	12	40	26.44	5.09

SD, standard deviations; PSSS, the Perceived Social Support Scale; SUPPH, the Strategies Used by People to Promote Health; CD-RISC-10, the 10-item Connor-Davidson Resilience Scale; LHS, Learned Helplessness Scale; **p < 0.01.

### Correlations between measures

Correlation analysis showed that LH was significantly negatively correlated with social support, individual resilience and self-efficacy. Furthermore, individual resilience and self-efficacy were positively correlated with social support (as shown in Table 3). The relationship between variables supports the test of subsequent hypotheses.

**Table 3:** Correlation coefficients between the variables.

Variables	1	2	3	4
1. PSSS	1			
2. SUPPH	0.304**	1		
3. CD-RISC-10	0.271**	0.305**	1	
4. LHS	−0.451**	−0.299**	−0.339**	1

PSSS, the Perceived Social Support Scale; SUPPH, the Strategies Used by People to Promote Health; CD-RISC-10, the 10-item Connor-Davidson Resilience Scale; LHS, Learned Helplessness Scale; **p < 0.01.

### Appropriateness of analysis criteria

The results of the Harman single factor test demonstrated that a total of 14 eigenvalues greater than 1 were extracted from principal component factor analysis. Among these factors, the maximum factor variance explanation rate was 35.04% (<40%), indicating no substantial CMB problem exists in the present research. VIF value = 1.147–1.171 (<5.0) and Cook’s distances = 0.000–0.178 (<1.0), which showed that there were no problem of serious multicollinearity and outliers in this study.

### Mediation effects of variables

**Figure 2:**
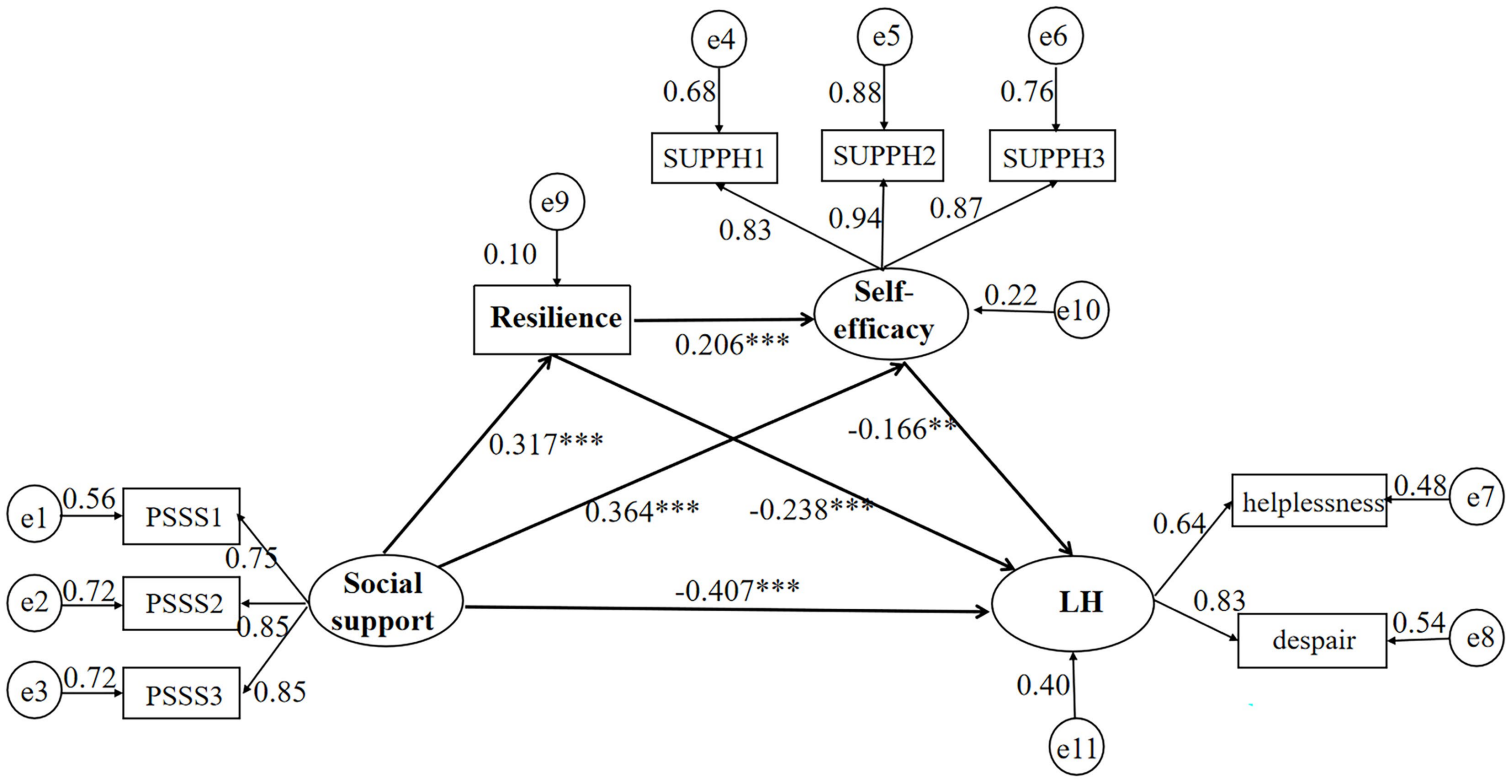
Chain mediating model of individual resilience and self-efficacy between social support and LH. **p < 0.01; ***p < 0.001. LH, learned helplessness; PSSS, the Perceived Social Support Scale; SUPPH, the Strategies Used by People to Promote Health; PSSS 1, friend support; PSSS 2, family support; PSSS 3, other important support; SUPPH 1, stress relief, SUPPH 2, positive attitude; SUPPH 3, self-decision.

The path model exhibited in Figure 2 comprised four latent variables (social support, resilience, self-efficacy, and LH) and eight observed variables. This model displayed a favorable fit with the data: *RMSEA* = 0.030, *SRMR* = 0.025, χ^2^/*df* = 1.473, *CFI* = 0.986, *NFI* = 0.958, *GFI* = 0.962, *TLI* = 0.983, and *IFI* = 0.986. As seen in Table 4, the effect of social support on LH was negatively and significantly mediated by individual resilience and self-efficacy. Further, the serial multiple mediation of both individual resilience and self-efficacy was statistically significant between social support and LH.

**Table 4:** Bootstrap analysis of the mediating effects of individual resilience and self-efficacy.

Effect	Standardized pathway	SE	95%CI	*p*
**Direct effects**				
H1: Social support → LH	−0.407	0.062	−0.510 to −0.308	0.000
Individual resilience → LH	−0.238	0.060	−0.337 to −0.139	0.000
Self-efficacy → LH	−0.166	0.055	−0.249 to −0.070	0.002
Social support → individual resilience	0.317	0.052	0.227–0.399	0.000
Social support → self-efficacy	0.364	0.047	0.281–0.438	0.000
Individual resilience → self-efficacy	0.206	0.053	0.119–0.292	0.000
**Indirect effects**				
H2: Social support → individual resilience → LH	−0.075	0.017	−0.083 to −0.027	0.001
H3: Social support → self-efficacy → LH	−0.060	0.015	−0.067 to −0.019	0.005
H4: Social support → individual resilience → self-efficacy → LH	−0.011	0.003	−0.015 to −0.003	0.003
**Total indirect effects**	−0.146	0.023	−0.142 to −0.066	0.001
**Total effects**				
Social support → LH	−0.553	0.051	−0.465 to −0.299	0.001

LH, learned helplessness; SE, standard error; CI, confidence interval.

## Discussion

The purpose of this study was to examine the structural model of LH based on social support: the mediating role of resilience and self-efficacy via SEM analyses. The results show that the conceptual model of the study is consistent with the data. It can be concluded that there is a significant relationship between these variables. The results of present study indicated that social support can significantly negatively predict LH in patients with lung cancer, which is in line with the view of previous research (Liu et al., 2024; Xie et al., 2023a). Hypothesis 1 is confirmed. Feeney and Collins (2015) put forward an integrative model of social support, demonstrating that social support can mitigate the deleterious effects of stressors through multiple channels, which include emotional states (e.g., minimizing negative impacts), appraisal of the events (e.g., positive appraisal), and context-specific behaviors (e.g., implementing effective coping strategies). Suffering from lung cancer inflicts a significant emotional trauma on patients, and social support serves as a crucial resource for these individuals in managing the impediments posed by the disease. Firstly, social support acts as a buffer between life adversities or hardships and psychological health (Cohen and Wills, 1985), which can buffer the deleterious effects of stress caused by lung cancer and its treatment, and help patients maintain a positive emotional experience, ultimately reducing their sense of LH. In the second place, a significant level of social support, encompassing but not limited to medical, emotional, and financial support, enables prompt patients to face the disease more optimistically, improve therapy compliance and implement effective symptom management strategies to maintain a health physical and mental condition (Şahin Altun et al., 2022), thereby mitigating their LH.

The current study suggested that individual resilience played a mediating role between social support and LH, and hypothesis 2 was verified. On the one hand, social support has a positive predictive effect on the individual resilience. As described above, the transactional model of resilience holds that environmental factors can enhance resilience by mitigating the deleterious impacts of stress on individuals (Richardson, 2002). Social support plays a vital role in furnishing individuals with crucial psychological resources by offering both emotional and material assistance, thereby effectively replenishing patients’ depleted mental energy and enhancing their individual resilience. A positive correlation was also detected between social support and individual resilience in breast cancer patients (Harmancı and Yıldız, 2024). On the other hand, individual resilience can negatively predicted LH. The function mechanism of individual resilience is not to entirely circumvent the external adverse effects of the environment, but rather to leverage personal strengths and available environmental resources to mitigate the intensity of crisis events (Masten et al., 2021). And this approach can break the cycle of negative impact, facilitate the growth of individual internal resources, and enhance the individual’s ability to confront challenges, adapt to setbacks, and achieve positive development (Li et al., 2020). Patients with lung cancer encounter significant physical and mental challenges throughout the diagnostic and treatment processes. The severity of symptoms, including persistent dry cough, sleep disruptions, compromised respiratory system function, fatigue, and the long-term struggles with chemotherapy-induced nausea and vomiting, radiation dermatitis, and other treatment side effects (Blaney, 2024), can invite feelings of helplessness and despair in patients. So, high resilient patients might be more inclined to employ their positive and optimistic attitude and tenacity to combat lung cancer, while concurrently conducting accurate cognitive assessments of the disease, thereby mitigating LH.

Supporting H3 of present study, we also discovered that self-efficacy negatively predicted LH, and mediated the association between social support and LH. This is similar to the conclusion of previous relevant literature, that is, social support significantly positively predicted self-efficacy (Chen Y. Y. et al., 2022), and self-efficacy significantly negatively predicted LH (Vergara et al., 2017). The possible reasons of positive association between social support are as follows: The Bandura self-efficacy theory (Bandura, 1977) posits that one’s self-efficacy can be established or fortified by significant external contributor, such as family function or social support. A higher level of social support correlates with increased social interactions and greater utilization of community health services (Lin et al., 2020). This, in turn, enables lung cancer patients to access more health-related information and acquire relevant skills for disease management, thereby enhancing their health self-efficacy. A research of 263 prostate cancer patients found that self-efficacy fully mediated the effect between social support and negative emotions with a 100% mediation rate (Wang et al., 2022). At the same time, the result confirmed that self-efficacy significantly negatively predicted LH. In accordance with Bandura (1977), self-efficacy governs an individual’s motivation to persist in the face of challenges by virtue of cognitive, motivational, emotional, and decision-making mechanisms. Hence, we can speculate that lung cancer patients with higher self-efficacy are good at self-attribution and adjustment, and characterizing setbacks as transient obstacles in the face of disease. As a result, they exhibit a higher degree of subjective initiative to improve health promotion behaviors and demonstrate a stronger determination to overcome related challenges, eventually leading to a decrease of LH. In addition, individuals with solid self-efficacy tend to exhibit effective emotional regulation (Gu et al., 2023). Consequently, they are more inclined to perceive satisfaction and experience a greater abundance of positive emotions and accordingly decrease their LH. In the cancer context, patients with elevated self-efficacy expectations demonstrate a more robust ability to manage cancer-related challenges, and report higher life quality and greater disease-adjustment compared to those with lower levels of self-efficacy (Du et al., 2022; Rha et al., 2022). To sum up, strong self-efficacy was related to lower degree of LH in patients with lung cancer.

As hypothesized, the chain mediating effect of individual resilience and self-efficacy was confirmed in this study, which validated the hypothesis 4. This research suggested that individual resilience can positively predict self-efficacy, which meant that lung cancer patients with greater resilience had a tendency to feel more confident and able to implement strategies promoting health. The association between individual resilience and self-efficacy verified in this study is in line with the conclusion of prior research (Chen C. et al., 2022; Navickienė and Vasiliauskas, 2023). Strength and optimism can significantly affect self-efficacy, bolster patients’ confidence and coping capacity when confronted with adverse situations, and facilitate the transformation of setbacks into valuable experience resources to mitigate negative experiences such as depression, and stigma and have a protective effect on depression (Wang L. et al., 2023). The journey of fighting against lung cancer is marked by moments of adversity and setbacks. Greater individual resilience enables patients to effectively manage challenging circumstances, facilitating the dismissal of negative self-perceptions and fostering greater confidence in health-promoting behaviors, thus reducing their sense of LH. In conclusion, the higher the social support of lung cancer patients, the higher the individual resilience, the better the self-efficacy, and ultimately the lower the generation and development of LH.

### Practical implications

In terms of theoretical significance, the current study provides evidence on the association between social support and LH in the lung cancer populations. We explored the chain-mediating effects of individual resilience and self-efficacy and tested new mediating pathways that could reveal the intrinsic association between social support and LH in lung cancer patients.

For the practical significance, by understanding the effect of individual resilience and self-efficacy on the association between social support and LH, health care providers can establish intervention programs targeting the LH among lung cancer patients. First of all, social support is a key protective factor of LH. Not only should medical personnel focus on the professional support provided within the hospital, but also pay attention to the support from family members, friends, and peer, in order to establish a robust social support system for patients. At the same time, helping patients enhance the identification and utilization of both material and emotional resources from relatives, friends, and medical professionals is also necessary. Secondly, the conclusion of this work emphasizes that both individual resilience and self-efficacy should be considered when intervening LH. On the one hand, it is essential for physicians and nurses to distinguish patients who have poor individual resilience and explore its associated factors, such as instable financial status and unfixed employment or retirement guarantees. And the interventions of increasing or stabilizing individual resilience, such as cognitive-behavioral therapy and mindfulness-based interventions (Walker et al., 2022), should be incorporated into routine cancer care. It is also suggested that conducting individual resilience interventions in conjunction with efforts to enact system-level changes targeted at adversities could potentially generate synergistic effects, amplifying any single intervention can have (Chen et al., 2024). On the other hand, health care providers are supposed to pay more attention to promote self-efficacy of lung cancer patients. Self-management programs, mobile applications, telehealth, gaming, and social media were found to enhance self-efficacy (Farley, 2020).

### Limitations and future perspectives

The several limitations of this study cannot be overlooked. First of all, a cross-sectional study design was employed in the current research, which prevents the determination of causal relationships between the four variables. To overcome this limitation, forthcoming research can apply longitudinal methodologies to deeply verify the hypothetical models from a dynamic perspective. Secondly, it is crucial to exercise caution when generalizing the results of this study, as the questionnaire survey was exclusively conducted at five tertiary hospitals in China. Thus, the cultural and contextual factors specific to this setting may influence the generalizability of our findings. Whether the models hypothesized in this study holds true for a wider range of lung cancer patients to be further tested. Last but not least, due to time limits, this study did not consider other potential variables. There may be other mediating factors in the association between social support and LH. In the future, the collective influence of multiple mediating variables can be taken into account.

## Conclusion

The present study investigated patients with lung cancer and included individual resilience and self-efficacy into the analysis to explore their mediating role between social support and LH. The analysis of the mediation effect suggested that, social support of patients with lung cancer might not only affect the level of LH directly, but also indirectly by individual resilience and self-efficacy as mediating variables. The mediation model included three mediation pathways: (1) the mediation path through individual resilience; (2) the mediation path through self-efficacy; and (3) the mediation path through both individual resilience and self-efficacy. Despite these limitations mentioned above, the present study contributed to a better understanding of the association between social support and LH, and the results emphasize the integrative role of individual resilience and self-efficacy as the underlying mechanism. The results of this investigation correspond with our theoretical frameworks, and also provide evidence that can form the basis for strategies that alleviate LH among lung cancer patients.

## Data Availability

The raw data supporting the conclusions of this article will be made available by the authors, without undue reservation.
